# Sleep as a Component of Health in Areas of Armed Conflict and Disaster

**DOI:** 10.7759/cureus.78559

**Published:** 2025-02-05

**Authors:** Bilal Irfan, Abdallah Abu Shammala

**Affiliations:** 1 Michigan Alzheimer's Disease Center, University of Michigan, Ann Arbor, USA; 2 Center for Bioethics, Harvard Medical School, Boston, USA; 3 Internal Medicine, European Gaza Hospital, Gaza, PSE

**Keywords:** comprehensive healthcare, conflict zone, disaster, sleep, war zones

## Abstract

Sleep is a vital physiological and psychological process essential for overall health and well-being, yet it is often severely disrupted in areas affected by armed conflict and disasters. In these environments, civilians frequently become internally displaced or refugees, experiencing fragmented or reduced sleep due to factors such as constant noise from drones and airstrikes, overcrowded shelters, and pervasive fear for personal safety. These sleep disturbances, including but not limited to insomnia and nightmares, are not merely secondary effects of a conflict or disaster but can also exacerbate stress responses, weaken immune function, and increase the risk of psychological toll and manifestations such as anxiety and depression. Public health initiatives increasingly recognize sleep disruption as both a predictor and consequence of trauma, highlighting the need for targeted interventions. However, the implementation of such interventions is often hindered by resource constraints in conflict and disaster zones. Elevated cortisol levels and inflammatory cytokines have been linked to prolonged sleep loss, which can further intensify stress responses and increase susceptibility to infections. Addressing sleep disturbances is crucial for improving overall health outcomes for the affected population and facilitating recovery and reintegration as crises subside. Integrating sleep-focused strategies into humanitarian and public health responses is essential for mitigating the long-term psychological and physical impacts of conflict and disaster, ultimately supporting the restoration of healthier and more stable communities.

## Editorial

Sleep is a physiological and psychological process fundamental to human health and well-being, yet it can be profoundly disrupted in areas affected by armed conflict and widespread disaster [1]. In areas where armed confrontations and chronic violence persist, sleep can become fragmented or significantly reduced. Civilians may be jolted awake by daily air-raid sirens or airstrikes that pierce through the night. People forced into shelters share cramped, unfamiliar spaces, such as tents, basements, or emergency housing. Refugees from war-torn areas also experience frequent nightmares. Individuals living under such conditions often suffer from repeated awakenings, hypervigilance, inability to fall or stay asleep, and even dread before attempting to sleep. Studies across various conflicts have shown that sleep disruptions are far from a trivial side effect of war; they potentially aggravate stress responses, weaken immune function, and exacerbate or even trigger mental health conditions such as anxiety, depression, and posttraumatic stress disorder (PTSD) [2,3].

Public health experts have increasingly recognized sleep disturbance as both a predictor and outcome of trauma. Insufficient or poor sleep can amplify the body’s stress response, disrupt immune function, fuel anxiety and depression, and increase vulnerabilities to conditions such as hypertension, glucose dysregulation, pain disorders, and hindered recovery from direct traumatic injuries [4]. At the same time, armed conflicts generate many factors that compound these harmful effects. The bright lights used by military forces at night, the sounds of bombs or drones buzzing overhead, the fear of sudden shelling, and the inability to maintain a regular sleep routine due to overcrowded shelters all contribute to making sleep a significant challenge. The heightened anxiety and the unpredictability of war may also lead to nightmares related to the day’s traumatic events. Even those far from the front lines, who do not experience direct bombardment, may suffer from nightmares or insomnia, sometimes triggered by persistent media coverage or the knowledge that loved ones or acquaintances are in imminent danger [3]. The concept of vicarious trauma illustrates how exposure to war extends beyond direct blasts or personal injuries.

Among conflict survivors, nightmares often involve vivid reconstructions of traumatic events, reimagined in horrifying ways that jolt individuals awake and perpetuate a cycle of vigilant insomnia. Studies on survivors of earthquakes and forced displacement events similarly reveal how trauma can manifest in dream content and degrade sleep quality [2,3]. In some cases, losing an entire night’s sleep becomes commonplace. At the personal level, restorative sleep helps stabilize mood, strengthen immune function, and reduce the severity of trauma-related symptoms such as nightmares or hypervigilance. This in turn can enhance day-to-day functioning and emotional resilience, both crucial for coping in hostile or uncertain environments. At the community level, populations that sleep better are more likely to engage cohesively, support one another, and participate effectively in collective rebuilding efforts. Indeed, prioritizing sleep can accelerate societal recovery by fostering clearer communication, increasing productivity, and fostering a stronger sense of hope, all of which are ingredients for restoring a sense of normalcy and stability once the immediate crisis has subsided.

Understanding how disruption of nightly rest drives health problems is critical for public health interventions. The role of the autonomic nervous system in PTSD may help explain how recurring nightmares keep arousal levels high throughout the night, hindering deep sleep stages. Without adequate deep sleep, the body cannot effectively modulate stress responses the following day. This can lead to sustained high blood pressure, weakened immune function, and an increased risk of secondary health complications [4]. Short or fragmented sleep can also worsen mood dysregulation, leading to irritability or aggression the next day, precisely the sorts of reactions that can further destabilize families and communities already under siege. At the population level, these effects can diminish resilience, hinder reintegration after forced migration, and place additional burdens on local healthcare systems. In a chain reaction, poor sleep can contribute to or exacerbate chronic headaches, increase susceptibility to seizures, or even accelerate neurodegenerative conditions through immune system changes. Airstrikes, missile bombardments, and prolonged armed conflicts highlight how large segments of the population, even those not directly injured, can develop serious sleep difficulties. Although property destruction, forced relocation, and bombardment are rarely seen as a sleep crisis, healthcare workers in active disaster or conflict zones have noted the frequent occurrence of insomnia and nightmares in both triage settings and formal psychological evaluations, when feasible.

Although not everyone with nightmares or insomnia meets the criteria for PTSD, sleep-specific symptoms can predict the development of more serious psychological consequences if left unaddressed. The uncertainty of schedules and displacement scenarios can disrupt normal circadian rhythms. Some individuals have reported sleeping in shifts, waking to watch over others, and missing large portions of nighttime rest for fear that if they do fall deeply asleep, they might not wake in time to flee. This sleep deprivation can accumulate quickly, with detrimental effects on cognition, reaction time, and emotional regulation.

Among conflict refugees in general, sleep disruptions stem not only from the immediate trauma but also from continuous changes in routine, nutrition, climate, indefinite waiting, and insufficient resources for basic comfort. Overcrowded refugee camps or shelters are often brightly lit around the clock. Noise levels remain high, and privacy is scarce. All in all, these conditions can make it extremely difficult to fall or stay asleep. Additionally, rates of depression, anxiety, and posttraumatic stress are often amplified by the ongoing chaos and insecurity, further disrupting sleep architecture.

While acute insomnia might initially reflect a normal reaction to intense stress or fear, it can evolve into chronic insomnia if the precipitating stressor remains unresolved or the body’s arousal levels remain elevated [4]. Over time, repeated nighttime awakenings, nightmares, or the dread of going to bed can become ingrained habits. People may also begin misusing sedatives in a desperate attempt to sleep, which can worsen sleep cycles. This underscores the importance of including direct attention to sleep in public health approaches to war-related trauma. Without a stable foundation of rest, individuals may be less responsive to psychosocial interventions or other therapies aimed at addressing trauma. Insomnia and other sleep disorders may not only predict the onset of PTSD but also intensify its severity. Interventions specifically targeting nightmares, such as imagery rehearsal therapy, which helps patients rewrite the script of their recurring nightmares, may be beneficial in diminishing these distressing nighttime experiences when possible to administer and, in turn, may aid in reducing the severity of daytime PTSD symptoms. Cognitive Behavioral Therapy for Insomnia (CBT-I) is another recommended approach for realigning disrupted circadian rhythms and addressing maladaptive sleep-related thoughts. However, access to specialized providers for CBT-I is often limited in conflict-affected populations [5]. Some small-scale local efforts have attempted to integrate elements of CBT-I with community-based trauma counseling or resource distribution programs to mitigate feelings of helplessness and reduce negative nighttime arousal. But the scale of the problem is immense when the entire population is affected.

Additionally, psychosocial resource loss, such as the breakdown of family structures, sudden unemployment, or displacement, places a unique strain on sleep by introducing worries about personal safety, finances, or the fate of displaced or missing relatives. Therefore, public health frameworks cannot afford to relegate sleep to the background. Different disaster and conflict zones present unique challenges, and what can be implemented as an intervention in environments with limited resources will vary. For millions of forcibly displaced persons, whether living in tents, using communal bathrooms, or sleeping in improvised vehicles, the capacity to improve the environment remains constrained. In these contexts, it becomes especially urgent for relief efforts to incorporate strategies that address sleep disruptions and educate impacted populations on the importance of sleep for health. Even partial solutions, such as ensuring that overhead lights are turned off at night, encouraging consistent wind-down routines, distributing warm blankets, or encouraging communal rotations to stand watch so that others can rest, can help mitigate the effects on circadian rhythms, when feasible in a given situation.

From a broader perspective, the intersection of disaster or conflict scenarios with sleep has far-reaching ramifications that can persist long after a specific event ends. If the general population’s sleep is impaired for months or years, entire communities may face elevated heightened psychological and physical impairments. This clarifies the importance of early screening for sleep problems during an intervention phase, be it for displaced individuals, war-injured patients, or even presumably lower-exposed groups in safer geographic zones who may still be indirectly affected. It also suggests that any post-conflict or post-disaster reconstruction strategy should incorporate sleep interventions aimed at improving living conditions, addressing nightmares and insomnia, and creating environments conducive to better sleep. By recognizing the importance of healthy sleep, humanitarian, clinical, and governmental responses can help mitigate the psychological toll on disaster- and conflict-affected populations and support their recovery process. Looking ahead, further research is essential to deepen our understanding of the relationship between sleep disruption, trauma, and long-term recovery in conflict and disaster contexts. Such research should explore the physiological mechanisms at play, as well as how poor sleep exacerbates mental and physical health burdens. Additionally, it should consider cultural factors, resource limitations, and how different stages of crisis influence outcomes. By identifying these key determinants and testing innovative, context-specific interventions, the field can move toward evidence-based solutions that effectively address sleep disturbances and optimize recovery for conflict- and disaster-affected populations.
